# Antimicrobial Usage in Horses: The Use of Electronic Data, Data Curation, and First Results

**DOI:** 10.3389/fvets.2020.00216

**Published:** 2020-04-29

**Authors:** Anne Schnepf, Astrid Bienert-Zeit, Hatice Ertugrul, Rolf Wagels, Nicole Werner, Maria Hartmann, Karsten Feige, Lothar Kreienbrock

**Affiliations:** ^1^Department of Biometry, Epidemiology and Information Processing, WHO Collaborating Centre for Research and Training for Health in the Human-Animal-Environment Interface, University for Veterinary Medicine Hannover, Hanover, Germany; ^2^Clinic for Horses, University for Veterinary Medicine Hannover, Hanover, Germany; ^3^Information and Data Service (TiHo-IDS), University for Veterinary Medicine Hannover, Hanover, Germany

**Keywords:** antimicrobial consumption, individual animal, electronic practice management software, Germany, antimicrobial resistance

## Abstract

The usage of antimicrobial drugs (AMs) leads to an increase in antimicrobial resistance (AMR). Although different antimicrobial usage (AMU) monitoring programs exist for livestock animals in Germany, there is no such system for horses. However, with the increasing usage of electronic practice management software (EPMS), it is possible to analyze electronic field data generated for routine purposes. The aim of this study was to generate AMU data for German horses with data from the Clinic for Horses (CfH), University of Veterinary Medicine Hannover (TiHo), and in addition to show that different processes of data curation are necessary to provide results, especially considering quantitative indices. In this investigation, the number of antimicrobial doses used and the amount and percentage of active ingredients applied were calculated. Data contained all drugs administered between the 1st of January and the 31st of December 2017. A total of 2,168 horses were presented for veterinary care to the CfH and 34,432 drug applications were documented for 1,773 horses. Of these, 6,489 (18.85%) AM applications were documented for 837 (47.21%) horses. In 2017, 162.33 kg of active ingredients were documented. The most commonly used antibiotic classes were sulfonamides (84.32 kg; 51.95 %), penicillins (30.11 kg; 18.55%) and nitroimidazoles (24.84 kg; 15.30%). In 2017, the proportion of Critically Important Antibiotics (CIA)—Highest Priority used was 0.15% (0.24 kg) and the proportion of CIA—High Priority used was 20.85% (33.85 kg). Of the total 9,402 entries of antimicrobial active ingredients, the three with the largest number used were sulfonamides [*n* = 2,798 (29.76%)], trimethoprim [*n* = 2,757 (29.76%)] and aminoglycosides [*n* = 1,381 (14.69%)]. Comparison between Administered Daily Dose (ADA) and Recommended Daily Dose of CfH (RDD_CfH_), showed that 3.26% of ADA were below RDD_CfH_, 3.18% exceeded RDD_CfH_ and 93.55% were within the range around RDD_CfH_. This study shows that data generated by an EPMS can be evaluated once the method is set up and validated. The method can be transferred to evaluate data from the EPMS of other clinics or animal species, but the transferability depends on the quality of AMU documentation and close cooperation with respective veterinarians is essential.

## Introduction

Antibiotics have a long history of usage in human and veterinary medicine. In 1910, the first antimicrobial compound arsphenamine was introduced (1), and in 1929, penicillin was discovered by Sir Alexander Fleming (2). Curing bacterial infections without severe side effects was an important milestone in the history of medicine.

Today, mankind is facing one of the biggest problems in treating bacterial infections: the increase of antimicrobial resistance (AMR) that is correlated with the increasing use of antimicrobials (3–6). In particular, multiple drug-resistant pathogens will cause an increasing number of deaths in humans and animals. Recently, published numbers from the European Center for Disease Prevention and Control (ECDC) showed that cases of death caused by resistant pathogens increased from an estimated 25,000 fatalities in Europe in 2007 (7) to 33,000 fatalities (8) in 2015.

The increasing occurrence of resistant bacteria is a controversial issue. From the One Health perspective, using antimicrobial drugs (AMs) in farm animals is often thought to facilitate the spread of AMR, because of the dissemination of resistant bacteria through the food chain. In general, the roles of horses and companion animals as facilitators of AMR have been underestimated (9) and received less attention (10–13).

Today, horses live in close contact with humans. There are an estimated 1.3 million horses in Germany (14); these horses could be seen as a reservoir and vector for resistant bacteria (15, 16).

Despite playing a role in the development of AMR, there is very little information about antimicrobial usage (AMU) in horses in Germany. Official reports on antimicrobials sold for veterinary use are based on data from the register of veterinary medicinal products and do not include reports on specific animals. Additionally, many drugs are authorized for multiple animal species, making an assignment to specific animal species impossible, and off-label use of human medicinal products is not included (17–23).

Therefore, it is vital to develop a system for collecting and analyzing data on the usage of antimicrobial drugs in horses to provide useful information for veterinarians. So the aim of the study was to generate AMU data under the system for German horses, and in addition to show that different processes of data curation are necessary to provide results, especially regarding quantitative indices.

## Materials and Methods

In Germany, AMs for animals are, by law (AMG §56a), only available with a prescription from a veterinarian, so data from clinics or practices offer a good basis for evaluating AMU.

Data from the Clinic for Horses, University of Veterinary Medicine Hannover, Foundation (TiHo), on drugs used within the study period between the 1st of January and the 31st of December 2017 were evaluated. These data were generated in the electronic practice management software (EPMS) easyVet [Veterinärmedizinisches Dienstleistungszentrum (VetZ) GmbH, Isernhagen, Germany].

Only horses that had been prescribed at least one drug within the investigated time period were included in the study.

The Clinic for Horses, which is a university hospital, works mainly as a referral hospital without out-patient care. There are 20 veterinarians with different levels of experience at the Clinic for Horses.

### Generating and Editing the Dataset

Data were extracted via export from easyVET. Extracted data were provided in Excel format (Microsoft, 2010) and imported into the statistical analysis software SAS 9.4 (SAS Institute Inc., Cary, NC, United States), where descriptive statistical calculations were performed.

For each horse, a unique animal identification (ID) number, breed, gender, date of birth, all documented weights, and status as food-producing animals were reported. For each drug, the following information was collected: treatment date, medicinal product name, amount and unit of the preparation and whether the drug was administered during the visit or dispensed to the owner. Further data collected were a unique case ID number and the corresponding diagnoses.

For this study, a few assumptions had to be made. First, it was assumed that all billed drugs were used to treat the horses, and only billed drugs were used. Second, it was assumed that only dosages based on a summary of product characteristics (SPC) and recent publications were applied and that every diagnosis verified by a veterinarian was documented in the system.

**Figure 1:**
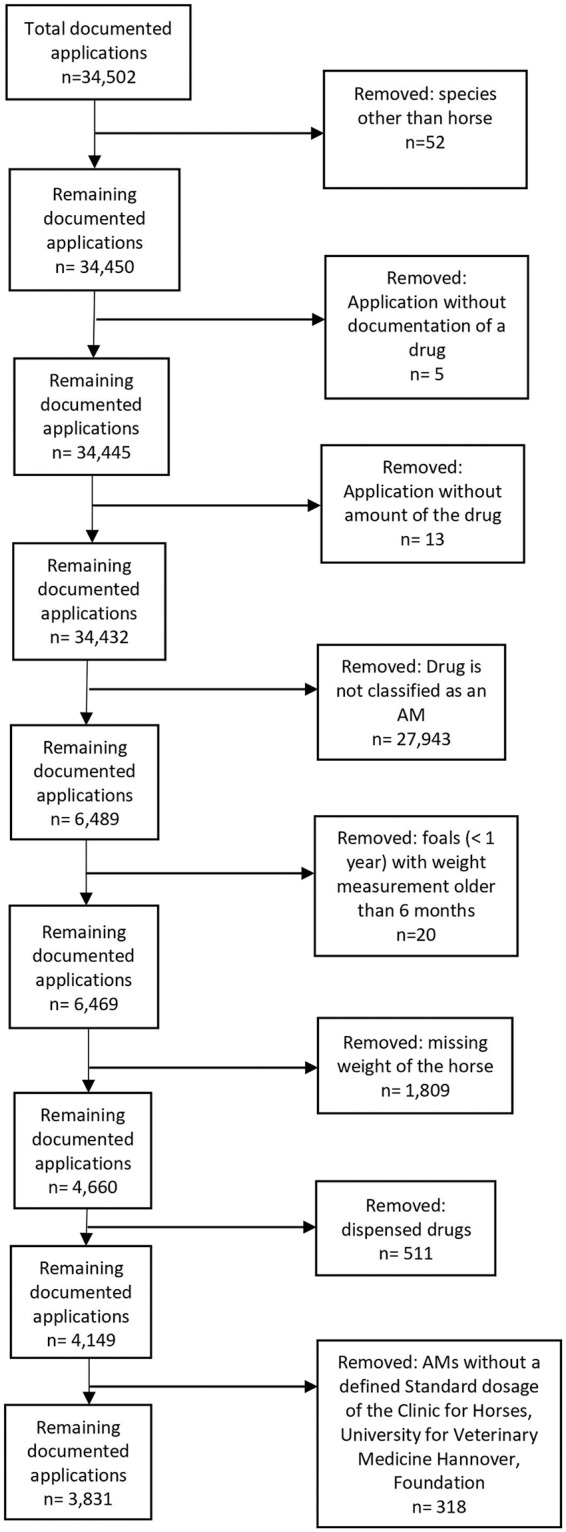
Data cleaning process for drug applications in 2017 at the Clinic for Horses, University for Veterinary Medicine Hannover, Foundation.

First of all, the following prescriptions were excluded (Figure 1): documented applications for species other than horse, documented applications without drug name, documented applications without amount of the drug.

Furthermore, in this study, AMs were defined as medicines that destroy or inhibit the growth of bacterial microorganisms (i.e., antibacterial drugs) and were authorized for systemic and topical use (24). Other AMs, such as antiviral or antifungal drugs or biocides, were excluded from this study (Figure 1).

A master table of AMs was developed using the product index of the clinic. All drug names were compared with different databases (vetidata.de, gelbe-liste.de, www.pharmnet-bund.de and drugs.com) to identify and extract all drugs containing at least one antimicrobial substance. In addition, products licensed for other species or humans, individually manufactured preparations and imported drugs were considered. The cascade principle [EU Regulation 37/2010, §56a (2), and in addition, §56a Abs. 2a AMG for equids] allows the off-label use of medicinal products not licensed for horses in cases where there is no alternative drug licensed for horses that would provide an appropriate treatment, or the drug is not authorized for the field of application.

If a product contained multiple active substances in the same preparation, substances other than antimicrobials were excluded from the calculations. Regarding amoxicillin plus clavulanic acid, only amoxicillin was included in the calculation, as clavulanic acid works as an adjuvant for penicillins and does not work as an antimicrobial by itself. In contrast, the quantity of sulfonamides and trimethoprim was calculated for each substance separately, as both are classified as antimicrobials. For any other compounded drug each substance by itself was included in the calculation with its own factor.

All antimicrobials were also categorized by their route of administration as per the SPC, resulting in three main groups: injection, oral and topical. The oral and topical routes were divided in several subgroups each (oral in tablets, capsules and oral—other; topical in eye, skin, ear and topical—other). Dividing injection into subgroups according to the exact route of administration (e.g., intravenously or intramuscular) was not possible with the extracted data or with the information given in the SPC.

Subsequently, the most recent WHO AM classification was applied: *Critically Important Antibiotics* (*CIA)—Highest Priority, CIA—High Priority, Highly Important* and *Important* (Table 1).

**Table 1:** Active ingredients documented to be used in horses in 2017 at the Clinic for Horses according to the World Health Organization (WHO) classification, antimicrobial group and chemical structure.

**WHO classification**	**Antimicrobial group**	**Substance**
CIA^*^–Highest priority	Cephalosporins	Cefquinome (4th generation)
	Quinolones	Enrofloxacin
		Marbofloxacin
		Moxifloxacin
		Ofloxacin
	Macrolides	Azithromycin
	Polypeptides	Polymyxin B
CIA^*^–High priority	Aminoglycosides	Amikacin
		Gentamicin
		Neomycin
	Ansamycins	Rifampicin
	Penicillins	Amoxicillin
		Benzylpenicillin
Highly important	Amphenicols	Chloramphenicol
	Sulfonamides	Sulfadiazine
		Sulfadimethoxine
		Sulfonamide
	Tetracyclines	Chlortetracycline
		Doxycycline
		Oxytetracycline
	Trimethoprim	Trimethoprim
Important	Nitroimidazoles	Metronidazole

* *CIA, Critically Important Antibiotics*.

To smoothen the analysis processes, species, breed, gender and drugs were numerically encoded. With this step, misspellings and repeated entries, such as multiple breeds or drugs, were consolidated.

### Choice of the Corresponding Diagnosis

Clinical diagnoses are manifold. To link the diagnosis and indication for antibiotic treatment, a decision tree based on the default catalog of diagnoses used by the Clinic for Horses was developed for choosing the diagnosis requiring AMU. Diagnoses in this catalog are composed of the affected organ system, detailed anatomic location, etiology and exact diagnosis. While developing this decision tree, primarily the etiology was used for choosing the diagnosis requiring AMU.

For cases where only one diagnosis was indicated, the single diagnosis was chosen for analysis. For cases with more than one diagnosis, choosing the diagnosis requiring for analysis was performed in an eleven-step process (see Supplement 1).

Using the unique animal ID number in combination with the unique case ID number, differentiation between recurring and distinct conditions was possible. The options for choosing a diagnosis in the EPMS can lead to different naming of the same condition; therefore, classification into distinct or recurring cases had to be performed manually.

### Calculation of the Administered Daily Dose (ADA)

To calculate the amount used for each active ingredient, the master table was compared to a database with all veterinary drugs officially licensed in Germany. The information of each product in this database contains at least a pharmaceutical form, a quantity unit, the possible routes of administration and the active ingredients with international nonproprietary name, agent group (by chemical structure), and amount of active ingredients per quantity unit.

With this information, an ADA of each active ingredient, in grams, was calculated by transferring the amount given for each course.

$$\begin{array}{l} ADA\;\left(Administered\;Daily\;Dose\right)=amount\;of\;drug\;\times \\ \;proportion\;of\;active\;ingredient\;in\;this\;drug \end{array}$$

### Recommended Daily Dose (RDD_CfH_) and Comparison With the ADA

The ADA of each active ingredient was compared to the Recommended Daily Dose internally defined by the TiHo Clinic for Horses (RDD_CfH_) based on the weight of the horse and standard dosages to define whether the ADA was below the RDD_CfH_, within a range around the RDD_CfH_ or exceeding the RDD_CfH_. Standard dosages (SD_CfH_) per kilogram per day were defined for each drug (see Supplement 2) with the information out of summary of product characteristics and recent research. For some active ingredients, such as amikacin, cefquinome, metronidazole, amoxicillin and ceftiofur, special dosages for foals were determined.

The calculated amount of active ingredient was defined as the RDD_CfH_ per animal for each active ingredient, and this value was calculated by multiplying the SD_CfH_ with the horses' weight measured at the closest date to treatment. If there was a weight entered before and after the day of treatment with the same time lag, the weight taken before treatment was chosen for analysis.

$$\begin{array}{l} RDD_{CfH}\;\left(Recommended\;Daily\;Dose\right)=SD_{CfH}\;\times \;bodyweight \end{array}$$

If SD_CfH_ consisted of a range of dosages and not a fixed value, the lowest and highest RDD_CfH_ were calculated with the lowest and highest SD_CfH_, respectively.

Comparing ADA with RDD_CfH_ was done to evaluate whether the administered dosage was acceptable, below RDD_CfH_ or above RDD_CfH_.

To compare ADA with RDD_CfH_ the ADA should be in the range of bioequivalence, e.g., from 80 to 125% of the RDD_CfH_. To adjust for anomaly cases, a higher dosage of up to 2-fold was assumed to be acceptable. If the RDD_CfH_ was indicated by a dose range, the acceptable range of the active compound was within 80% of the lowest RDD_CfH_ and 125% of the highest RDD_CfH_, considering the range of bioequivalence, i.e., ADAs between 80% of the lowest RDD_CfH_ and 250% of the highest RDD_CfH_ were defined as acceptable doses. If RDD_CfH_ consisted a fixed value, ADAs between 80 and 250% of this value were defined as acceptable doses.

In the dataset, certain drugs could be administered multiple times per day. Therefore, these drugs had to be classified differently on the first and last day of application, as the exact time of arrival and discharge or surgery were unknown. If a total given amount on the first and last day was lower than the RDD_CfH_, it was assumed that, due to the time of arrival or discharge, a full RDD_CfH_ was not possible. Therefore, we applied the same adjustment used for the range of bioequivalence for proportions of the daily dose on the first and last day of treatment for each drug.

For the calculation of the RDD_CfH_ and comparison with the ADA, only a part of the dataset could be used (Figure 1). Entries without a weight had to be excluded because calculation of the RDD_CfH_ and ADA was not possible. To avoid a misclassification, only drugs administered were evaluated, and entries of foals with a weight measurement that was older than 6 months were excluded. Only drugs where the SD_CfH_ was available and calculating the RDD_CfH_ was possible were included. In total, 2,658 entries (40.96%) were excluded; the majority of these entries (1,809 entries; 27.88%) were excluded because of missing weight.

## Results

In 2017, 2,168 horses were presented to the study clinic. Of these 1,733 (81.78%) horses had at least one documented drug application and 837 (38.60%) horses received at least one AM.

A total of 34,432 drug applications were documented for 1,733 horses in the study clinic in 2017. Of these 34,435 drug applications, 6,489 were AMs administered to 837 horses. Thus, 18.85% of all drugs given were AMs, and 47.21% of all treated horses received at least one AM. There were 43 different antimicrobial drugs with 22 different active ingredients used. The active ingredients were classified into twelve groups by their chemical structure and into four groups based on WHO classification (Table 1).

In this study, the cascade principle was used for eight veterinary drugs not licensed for horses, for 22 drugs only licensed for humans and for two drugs individually manufactured.

The 6,489 AM applications were split into 3,316 (51.10%) oral AM applications, 2,799 (43.13%) injections, and 374 (5.76%) topical AM applications. Seven hundred and fourteen (11.00%) of the AM applications were dispensed, and 5,774 (89.00%) were used for treatment in the clinic.

Using the unique case ID number, we determined that drugs were used in 2,178 different cases and, in 914 (41.97%) cases among 837 horses, AMs were prescribed. Fifty-nine (7.05%) of 837 horses had more than one case ID number, meaning that they were represented multiple times. Of these horses, 49 (83.05%) represented two cases, and ten (16.95%) horses represented three or more different cases; there was a maximum of seven cases per animal. Fifteen (25.42%) of the 59 horses were treated with antimicrobials because of distinct conditions, while 40 (67.80%) were treated for recurring conditions. Four horses (6.78%) had different case ID numbers because of recurring and distinct conditions.

In 585 (64.00%) of 914 cases, the animal was defined as a non-food-producing horse, while 103 (11.27%) were defined as food-producing horses. In 226 (24.73%) cases, the status was either unknown or was not entered into the system.

In 2017, a single AM was administered in 51.64% (*n* = 472) of the cases, two different AMs were administered in 22.65% (*n* = 207) three AMs were administered in 17.61% (*n* = 161), and between four and eight different AMs were administered in 8.1% (*n* = 74).

In total, 162.33 kg of antimicrobial ingredients were administered or dispensed in 2017 (Table 2). Sulfonamides (84.32 kg; 51.95%) had the largest share, with sulfadiazine (56.29 kg; 66.76%) used most often. Sulfonamides were administered mostly orally (83.94 kg; 99.55%). Penicillins (30.11 kg; 18.55%) were in second place in the ranking of the amount of active ingredients used. Benzylpenicillin was the penicillin used most often (20.14 kg; 66.89%), and injection was the main route of administration (29.66 kg; 98.51%). The 3rd place position was taken by nitroimidazoles with metronidazole as the only active ingredient used in this AM group. In total, 24.84 kg (15.30%) of nitroimidazoles were administered, mostly via the oral drug route (24.79 kg; 99.80%).

**Table 2:** Documented amount of antimicrobial active ingredients used in horses in 2017 at the Clinic for Horses, by route of administration.

**Antimicrobial group and active ingredient**	**Injection Amount in kg**	**Oral Amount in kg**	**Topical Amount in kg**	**Total amount in kg (%)**
**Aminoglycoside**	**3.62**	–	**0.00**	**3.63 (2.23%)**
Amikacin	0.19	–	–	0.19 (0.11%)
Gentamicin	3.44	–	0.00	3.44 (2.12%)
Neomycin	–	–	0.00	0.00 (0.00%)
**Ansamycins**	–	**0.11**	–	**0.11 (0.07%)**
Rifampicin	–	0.11	–	0.11 (0.07%)
**Penicillins**	**29.66**	**0.45**	–	**30.11 (18.55%)**
Amoxicillin	9.53	0.45	–	9.98 (6.15%)
Benzylpenicillin	20.14	–	–	20.14 (12.40%)
**Cephalosporin**	**0.03**	–	–	**0.03 (0.02%)**
Cefquinome	0.03	–	–	0.03 (0.02%)
**Amphenicol**	–	–	**0.00**	**0.00 (0.00%)**
Chloramphenicol	–	–	0.00	0.00 (0.00%)
**Quinolones**	**0.05**	**0.01**	**0.00**	**0.07 (0.04%)**
Enrofloxacin	0.01	0.01	–	0.02 (0.01%)
Marbofloxacin	0.04	–	–	0.04 (0.03%)
Moxifloxacin	–	–	0.00	0.00 (0.00%)
Ofloxacin	–	–	0.00	0.00 (0.00%)
**Macrolide**	–	**0.06**	–	**0.06 (0.04%)**
Azithromycin	–	0.06	–	0.06 (0.04%)
**Nitroimidazole**	**0.04**	**24.79**	**0.00**	**24.84 (15.30%)**
Metronidazole	0.04	24.79	0.00	24.84 (15.30%)
**Polypeptide**	**0.08**	–	**0.00**	**0.08 (0.05%)**
Polymyxin-B	0.08	–	0.00	0.08 (0.05%)
**Sulfonamide**	–	**83.94**	**0.38**	**84.32 (51.95%)**
Sulfadiazine	–	56.11	0.18	56.29 (34.67%)
Sulfadimethoxine	–	27.84	–	27.84 (17.15%)
Sulfonamide	–	–	0.20	0.20 (0.12%)
**Tetracycline**	**0.00**	**2.29**	**0.00**	**2.30 (1.42%)**
Chlortetracycline	–	–	0.00	0.00 (0.00%)
Doxycycline	–	2.29	–	2.29 (1.41%)
Oxytetracycline	0.00	–	0.00	0.00 (0.00%)
**Trimethoprim**	–	**16.78**	–	**16.78 (10.34%)**
Trimethoprim	–	16.78	–	16.78 (10.34%)
**Total**	**33.50**	**128.44**	**0.39**	**162.33 (100.00%)**

–*, observed zero; 0, zero by rounding. The bold values are the summary per antimicrobial group, the corresponding active ingredients are underneath*.

Of the total 9,402 entries, the three active ingredients with the largest amount used were sulfonamides [2,798 entries (29.76%)], penicillins [1,362 entries (14.49%)], and nitroimidazoles [292 entries (3.11%)] (Table 3). The ranking of drugs used by the number of entries showed sulfonamides in first place with 2,798 entries (29.76%), trimethoprim in second place with 2,757 entries (29.32%) and aminoglycosides in third place with 1,381 entries (14.69%).

**Table 3:** Documented number of antimicrobial active ingredients used in horses in 2017 at the Clinic for Horses, by route of administration.

**Antimicrobial group and active ingredient**	**Injection**	**Oral**	**Topical**	**Total documented applications (%)**
**Aminoglycosides**	**1,222**	**–**	**159**	**1,381 (14.69%)**
Amikacin	141	–	–	141 (1.50%)
Gentamicin	1,081	–	3	1,084 (11.53%)
Neomycin	–	–	156	156 (1.66%)
**Rifamycin**	**–**	**70**	**–**	**70 (0.74%)**
Rifampicin	–	70	–	70 (0.74%)
**Penicillins**	**1,312**	**50**	**–**	**1,362 (14.49%)**
Amoxicillin	1,094	50	–	1,144 (12.17%)
Benzylpenicillin	218	–	–	218 (2.32%)
**Cephalosporins**	**61**	**–**	**–**	**61 (0.65%)**
Cefquinome	61	–	–	61 (0.65%)
**Amphenicols**	**–**	**–**	**3**	**3 (0.03%)**
Chloramphenicol	–	–	3	3 (0.03%)
**Quinolones**	**27**	**1**	**57**	**85 (0.90%)**
Enrofloxacin	4	1	–	5 (0.05%)
Marbofloxacin	23	–	–	23 (0.24%)
Moxifloxacin	–	–	53	53 (0.56%)
Ofloxacin	–	–	4	4 (0.04%)
**Macrolides**	**–**	**52**	**–**	**52 (0.55%)**
Azithromycin	–	52	–	52 (0.55%)
**Nitroimidazoles**	**8**	**242**	**42**	**292 (3.11%)**
Metronidazole	8	242	42	292 (3.11%)
**Polypeptide**	**168**	**–**	**156**	**324 (3.45%)**
Polymyxin-B	168	–	156	324 (3.45%)
**Sulfonamide**	**–**	**2,757**	**41**	**2,798 (29.76%)**
Sulfadiazine	–	1,672	25	1,697 (18.05%)
Sulfadimethoxine	–	1,085	–	1,085 (11.54%)
Sulfonamide	–	–	16	16 (0.17%)
**Tetracyclines**	**1**	**144**	**72**	**217 (2.31%)**
Chlortetracycline	–	–	62	62 (0.66%)
Doxycycline	–	144	–	144 (1.53%)
Oxytetracycline	1	–	10	11 (0.12%)
**Trimethoprim**	**–**	**2,757**	**–**	**2,757 (29.32%)**
Trimethoprim	–	2,757	–	2,757 (29.32%)
**Total**	**2,799**	**6,073**	**530**	**9,402 (100.00%)**

–*, observed zero; 0, zero by rounding. The bold values are the summary per antimicrobial group, the corresponding active ingredients are underneath*.

Drugs licensed for horses had the largest share of the total amount of active ingredient used, with an amount of 109.83 kg (67.66%). The amount of drug used that was not licensed for horses was 52.50 kg (32.34%). Of the drugs not licensed for horses, 25.38 kg (48.34%) were originally licensed for humans, 2.73 kg (5.20%) were licensed for other animal species and 24.39 kg (46.46%) were individually manufactured. Out of the drugs licensed for human use only, the largest proportions were observed for benzylpenicillin (20.14 kg; 79.35%) and gentamicin (3.44 kg; 13.55%).

**Figure 2:**
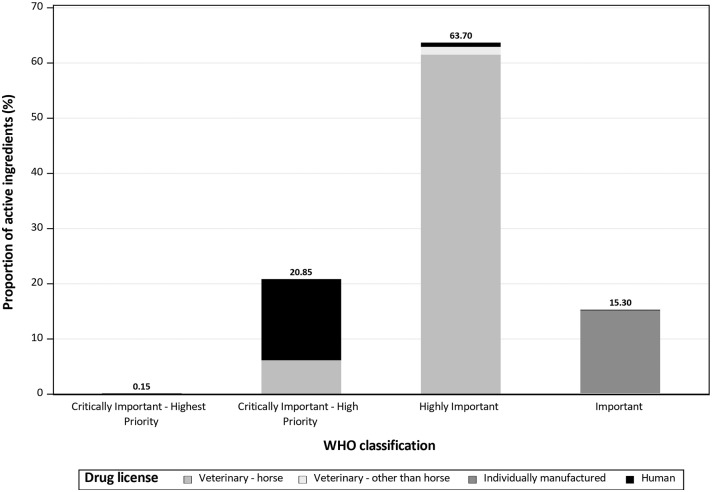
Proportion of the amount of antimicrobial active ingredients reported to be used in horses in 2017 at the Clinic for Horses, University for Veterinary Medicine Hannover, Foundation, by drug license type and World Health Organization classification.

Referring to the WHO classification, 0.24 kg (0.15%; see Figure 2) of the drugs used were classified as *CIA—Highest Priority*; the drugs used in this category were mostly drugs licensed for animals other than horse, and polymyxin B as main active ingredient. Overall, 33.85 kg (20.85%) were classified as *CIA—High Priority*. Most of these drugs were licensed for humans, and benzylpenicillin accounted for the largest proportion. In the group classified as *Highly Important* (103.41 kg; 63.70%), the largest proportion of AM drugs used were licensed for horses, and sulfadimethoxine was the main active ingredient. Individually manufactured drugs provided the largest proportion of drugs classified as *Important* (24.84 kg; 15.30%), with metronidazole as the most common main active ingredient.

The comparison between the ADA and RDD_CfH_ showed that, of the 3,831 drug applications where the comparison was possible, only 125 drug applications (3.26%) were below the RDD_CfH_, 122 (3.18%) drug applications exceeded the RDD_CfH_ and 3,584 (93.55%) drug applications were within the range around RDD_CfH_.

## Discussion

Because of the increasing resistance of bacteria against antimicrobial compounds, there is a need to collect and evaluate data on AMU in companion animals such as horses and pets. To the best of our knowledge, this is the first study investigating the usage of antimicrobials in an equine clinic in Germany. Until now, such information has been scarce. Sales data of veterinary medicinal products are published annually (17), but these data do not refer to the species that the drug is used for, and off-label use of medicinal products licensed for humans or other animal species and individually manufactured AMs are not taken into account.

In this retrospective study, we used EPMS data that display the real usage of all kinds of antibacterial drugs. These large-scale data are generated within routine clinical work and do not require additional efforts from the veterinary practitioner involved. However, close cooperation with the respective veterinarians is crucial for a realistic evaluation, as errors in documentation are possible and can be found more easily when working with a close contact. In particular, when defining a standard dosage for each AM and choosing a corresponding diagnosis, knowledge in clinical work is essential.

This study presents the broad possibilities and benefits of analyzing data generated by EPMS, but also acknowledges the assumptions and adjustments that had to be made in advance.

In general, the results show that 47.21% of all horses treated with at least one drug, received at least one antimicrobial, but there was a very low frequency and amount of drugs classified as *CIA—Highest Priority* (0.15%; 0.24kg). The AMs used most often were classified as *Highly Important*, and the biggest proportion of the AMs used were licensed for horses.

Due to the lack of data, comparison of results with other studies or monitoring systems is difficult and only possible with reservations. Buckland et al. (25) showed that, in general, using EPMS data to study AMU in small animals is possible, but their way of evaluation is not applicable in our study because of the differences in investigated animal species between the studies and in the data that can be extracted from the particular EPMS. Furthermore, Buckland et al. (25) were working with a free text search to extract relevant treatment records, and it was only possible for these authors to extract a unique patient ID number (not a case ID number). As we were able to extract the drug name from easyVET, the source of errors related to AMU missing entries could be neglected in our study, while a free text search always bears a certain risk of drug misallocation. Therefore, these procedures are prone to information bias. In our study, it was possible to differ between prescriptions written for repeated vs. distinct conditions through the unique case ID number, which reduces this bias.

In our research in 41.97% of cases at least one AM was prescribed, which is comparable to results from Redding et al. (26) were AMs were prescribed in 38.4% of visits. Both proportions seem to be closely connected, but due to different definitions of cases and visits, detailed comparison is not possible.

In general, it can be said that correctness of the documentation performed by the veterinarians is crucial in both methods. As clinical routine data are used for evaluating errors in documentation, plausibility checks are needed to reduce these errors to a minimum.

Schwechler (27) undertook a theoretical exercise where they asked veterinarians from Germany, Austria, and Switzerland which kind of AM they would prescribe in six different given hypothetical clinical cases in equine medicine.

The participants stated that they would prescribe cephalosporins of the 3rd and 4th generation in 11% of the hypothetical cases, and fluoroquinolones in 4% of the cases (27). These proportions take the results from Germany, Switzerland and Austria into account (27).

Depending on their *in vitro* spectrum of activity, cephalosporins can be categorized into four generations. The 3rd generation is composed among others of the active ingredients ceftiofur and ceftriaxone, the 4th generation of cefquinome and cefepime (28).

The authors also noted that in private equine clinics, cephalosporins of the 3rd and 4th generation are theoretically more often prescribed than in clinics of universities and private practice.

Results from De Briyne et al. (29) showed a similar theoretically prescription for horses in Germany. They asked veterinarians to name the five indications where AMs were prescribed the most and which group of AMs they would prescribe. Over all indications cephalosporins of the 3rd and 4th were mentioned in 9% and fluoroquinolones in 4% of theoretically prescriptions.

The results of this study show that only 0.65% of the drug applications at the Clinic for Horses were cephalosporins of the 4th generation. There was no application of cephalosporins of any other generation in the Clinic for Horses in 2017, as shown in Table 1. Fluoroquinolones were used in 0.90% of drug applications. There was a considerably lower usage of cephalosporins and fluoroquinolones in the Clinic for Horses than in the study of Schwechler (27) and in the study of De Briyne et al. (29).

In 2017, only a small amount (0.22 kg) of active ingredients with the WHO classification *CIA—Highest Priority* was used. This highlights that usage of these AMs was avoided, and *Highly Important* AMs were chosen instead. AMs licensed for horses were preferred (Figure 2). Active ingredients classified as *CIA-Highest Priority* were mostly used for specific ocular diseases. An exception is polymyxin B which is also classified as *CIA-Highest Priority*, but depending on the drug and indication, it is used either topically or injected. Injections were mainly administered during or directly after colic surgery in horses with signs of endotoxemia.

More detailed results with linkage between diagnoses and AMU could not be provided as documentation of diagnoses is not uniform between veterinarians.

Furthermore, the results from Schwechler (27) showed that 12% of the prescribed dosages were below the dosage recommended by the SPCs, and 72% of prescribed dosages were too low compared to dosages recommended in recent publications. The authors criticized that the recommended dosages of the SPCs were often obsolete and did not relate to recent publications.

In another study, Hughes et al. (30) sent a questionnaire to veterinarians in the United Kingdom and revealed that 5.8% of theoretical prescriptions were underdosed and 56.9% were overdosed compared to dosages issued by the VMD.

In the study from Schwechler (27) and the study from Hughes et al. (30) only the recommended dosages themselves were defined as acceptable dosages.

For comparison of the results of Schwechler (27), Hughes et al. (30) and the results of this study, it must be considered that the results of this study are based on routine treatment data and not theoretically prescribed AMs. It also has to be noted that our study was based on data generated for accountancy and documentation purposes and not for research purposes. Therefore, the data must be examined for implausibility.

Possible sources for errors were not only falsely documented amounts of used AMs, but also outdated weights of treated animals, where the actual weight was used to calculate the dose but not entered into the system. Another possible source for errors was drugs falsely documented as being used in the clinic instead of being handed over to the animal owner. This led to a higher calculated ADA, as an amount for a few days dispensed to the owner was wrongly entered as a single treatment. Plausibility checks were performed, and a very high proportion of plausible data was found.

Dosages based on the SPCs and recent publications were used to define the SD_CfH_. A mixture of both was used to formulate a realistic classification while comparing ADA to RDD_CfH_, as recommended dosages in the SPCs could change after authorization because of recent research.

When comparing the UDD of the Clinic for Horses in 2017 to the RDD_CfH_, there was very little deviation. Investigations of prescriptions of the Clinic for Horses in 2017 showed that with 3.26% of all entries below the RDD_CfH_ and 3.18% above the RDD_CfH_, there was a high level of responsibility used when choosing the correct dosages.

In this study, calculation of the ADA was not possible for 1,809 entries because of missing weights, and these entries were excluded. For this study, the results of the comparison between the ADA and RDD_CfH_ need to be interpreted with caution, but it is assumed that missing bodyweights are purely by chance, and therefore, a change of proportions in the results is possible in both directions. It is assumed that this did not lead to a selection bias.

In total, 733 t of antimicrobial active ingredients were sold to the veterinary sector in Germany in 2017 (31). Here, the biggest proportion falls upon penicillins (36.7%), which was twice as much as the proportion used in the Clinic for Horses. Tetracyclines had the second biggest share with 25.94%. In contrast, in the Clinic for Horses, only 1.42% of the total amount used was tetracyclines. The difference in polypeptides is also obvious: in 2017, 10.1% of the total amount of active ingredients sold belonged to this group, while only 0.08% of the amount administered by the Clinic for Horses did.

To evaluate the results of the annual sales data in comparison to the amounts used in the Clinic for Horses, it is important to know that the biggest proportion of drugs sold is licensed for farm animals and only a relatively small amount was licensed for horses (32). Moreover, because of multiple approvals, an assignment to one species is not possible for most of the drugs. Additionally, off-label use is not considered in the sales data. In our study we could show, that off-label use should not be neglected: in the Clinic for Horses, 32.33% of the total amount of drugs used were not licensed for horses and, thus, affected the proportion and amount of active ingredients used.

As previously mentioned, there are different official and private monitoring systems in Germany for AMU in livestock [Herkunftssicherungs- und Informationssystem für Tiere (33); QS Qualität und Sicherheit GmbH (34)]. Both systems use information from official German Application and Delivery Forms (ADF) for evaluating AMU in livestock. An ADF must be filled out every time a food-producing animal is treated with a drug. In the European Union (EU), horses can be declared as food-producing or non-food-producing animals in the equine passport, and ADFs are mandatory only for food-producing horses. As long as it is not stated otherwise, horses count as food-producing animals, and only a limited number of antibacterial drugs can be used. In particular, the application of certain AMs, such as chloramphenicol, dapsone, dimetridazole, metronidazole, nitrofurans, and ronidazole, is prohibited by European law (35).

The status of a horse can be changed from food-producing to non-food-producing at any time to extend the possibilities of treatment, but once it is changed, it can never be withdrawn. A system using data from ADF can only be used for food-producing horses because these forms are only mandatory for animals entering the food chain. Furthermore, existing monitoring systems use treatment frequency for comparing AMU in different production types that relate to the reference population on the farm. In contrast, treatment in horses is always individualized, with a treatment plan for each single horse. Thus, transferring existing systems to horses is not possible, and a system to evaluate data on AMU in horses must be developed instead.

In 2017, 64.00% (*n* = 585) of the cases in the Clinic for Horses were non-food-producing horses, while 36.00% (*n* = 329) were treated as food-producing animals. Consequently, using the existing systems based on ADFs, only 1/3 of cases and the related drug applications in the Clinic for Horses in 2017 would have been taken into account, and the results would not have captured the real AMU picture in horses.

A comparison to other international reports, and therefore, a more detailed estimation of the consequences of using data from only food-producing horses is not possible, as these reports do not take the status of the horses into account (19).

Due to a series of missing values and dynamic changes in the status of food or non-food animals, presentation of analysis about AMU grouped by the status of the horse is not possible.

As a general rule, it can be stated that depending on the method of selecting data from an EPMS and the extent of these data, the developed method can not only be adapted to other species but also to data from EPMS other than easyVET. As EPMS always work as an accounting tool, information about the used amount of an AM is entered and can be used for calculating total amounts of active ingredients used. Other possible evaluations within this system are those in conjunction with the WHO classification, route of administration and the comparison between the ADA and RDD. The feasibility of evaluating diagnoses associated with the active ingredients used depends on the method of documentation of the diagnosis. If chosen from a given catalog, as in our study, the developed method can be used. If diagnoses are entered as free text, using this information in further investigations requires more effort, and a free text search has to be applied. This method bears certain risks; misspellings, abbreviations and special terms have to be taken into account, which increases the possibility of an information bias. However, easyVET is used in 5,000 clinics and practices worldwide (36), and therefore, large-scale data can be evaluated easily, as only the drugs used and the standard dosages have to be adapted.

For the corresponding clinics or practices, this evaluation enables valuable feedback on AMU and provides a baseline regarding AMU. Once a baseline has been set, data can be compared to it continuously, and changes in prescription habits can immediately be investigated. Thus, the developed method supports adherence to guidelines on antimicrobial usage in clinics and practices and facilitates compliance with standards such as good veterinary practice and quality management systems. The results can also be used for antimicrobial stewardship programs because the results of the developed method can illustrate room for improvement.

To better understand and combat AMR in the future, more validated information on AMU in different animal species is needed. It is vital to monitor and analyze data on AMU continuously, especially regarding the transmission of resistant bacteria between animals and humans. The method applied in our study offers a tool to monitor AMU not only in horses but also in other animal species, and it could facilitate the desired reduction in AMU. Because of the changing legal requirements in the EU about documenting AMU for all animal species, including companion animals, a tool for evaluating clinical routine data about AMU is needed (37).

## Conclusions

Because of the threat of increasing AMR, it is crucial to make data about AMU available to preserve antimicrobial therapies. EMPS provides the possibility to extract large-scale data for analyses of AMU in clinics. Additionally, the corresponding veterinarians benefit without putting much effort into it, as the results provide very useful information that helps to improve their clinical work, such as adherence to guidelines on antimicrobial usage. In addition, this analysis allows comparison with AMU data from other animal species. Further investigations of AMU in combination with results of antimicrobial susceptibility testing, as well as analyses of data from clinics across the country, are necessary to provide a representative picture of AMU in horses in Germany.

## Data Availability Statement

The data were made available through internal cooperation. Therefore, any data transfer to interested persons is not allowed without an additional formal contract. Data are available to qualified researchers who sign a contract with the University of Veterinary Medicine Hannover. This contract will include guarantees to the obligation to maintain data confidentiality in accordance with the provisions of the German data protection law. A data access committee will be established on demand. This committee will consist of the authors as well as members of the University of Veterinary Medicine Hannover. Interested cooperative partners, who are able to sign a contract as described above, may contact: Lothar Kreienbrock, Department of Biometry, Epidemiology and Information Processing, University of Veterinary Medicine, Hannover, Bünteweg 2, 30559 Hannover, Email: lothar.kreienbrock@tiho-hannover.de.

## Ethics Statement

The data used in this study are based on data generated for accountancy and documentation purposes. Our research does not involve any regulated animals, and there were no scientific procedures performed on animals of any kind. For this reason, formal approval by an ethical committee was not necessary under the provisions of the German regulations.

## Author Contributions

AS and LK: conceptualization, formal analysis, investigation, methodology, and writing—original draft. AS, HE, RW, and MH: data curation. AS: project administration and validation. AS, HE, and MH: software. LK: supervision. AS, NW, AB-Z, KF, and LK: writing—review and editing.

## Conflict of Interest

The authors declare that the research was conducted in the absence of any commercial or financial relationships that could be construed as a potential conflict of interest.

## Supplementary Material

https://www.tiho-hannover.de/kliniken-institute/institute/bioepi/publikationen/zusatzmaterial-publikationen/

## Abbreviations

ADA(s): Administered Daily Dose(s)
ADF: Application and Delivery Forms
AM(s): Antimicrobial Drug(s)
AMG: Medicinal Products Act (“Arzneimittel-Gesetz”)
AMR: Antimicrobial Resistance
AMU: Antimicrobial Usage
CIA(s): Critically Important Antibiotic(s)
ECDC: European Centre for Disease Prevention and Control
EPMS: Electronic Practice Management Software
EU: European Union
ID number: Identification number
RDD_CfH_, Recommended Daily Dose of the Clinic for Horses: TiHo
SD_CfH_, Standard Dosage of the Clinic for Horses: TiHo
SPC: Summary of Product Characteristics
TiHoUniversity for Veterinary Medicine Hannover: Foundation (Stiftung Tierärztliche Hochschule Hannover)
WHO: World Health Organization.
